# Design and technical validation to generate a synthetic 12-lead electrocardiogram dataset to promote artificial intelligence research

**DOI:** 10.1007/s13755-023-00241-y

**Published:** 2023-08-30

**Authors:** Hakje Yoo, Jose Moon, Jong-Ho Kim, Hyung Joon Joo

**Affiliations:** 1 Korea University Research Institute for Medical Bigdata Science, Korea University College of Medicine, Seongbuk-gu, Seoul, Republic of Korea; 2 Department of Bio-Mechatronic Engineering, Sungkyunkwan University College of Biotechnology and Bioengineering, Jangan-gu, Suwon, Gyeonggi Republic of Korea; 3 Medical AI Research Center, Research Institute for Future Medicine, Samsung Medical Center, Gangnam-gu, Seoul, Republic of Korea; 4 Department of Cardiology, Cardiovascular Center, Korea University College of Medicine, Seongbuk-gu, Seoul, Republic of Korea; 5 Department of Medical Informatics, Korea University College of Medicine, Seongbuk-gu, Seoul, Republic of Korea

**Keywords:** Synthetic ECG dataset, Public database, Deep learning, 12-lead ECG, Cardiovascular disease

## Abstract

**Purpose:**

The purpose of this study is to construct a synthetic dataset of ECG signal that overcomes the sensitivity of personal information and the complexity of disclosure policies.

**Methods:**

The public dataset was constructed by generating synthetic data based on the deep learning model using a convolution neural network (CNN) and bi-directional long short-term memory (Bi-LSTM), and the effectiveness of the dataset was verified by developing classification models for ECG diagnoses.

**Results:**

The synthetic 12-lead ECG dataset generated consists of a total of 6000 ECGs, with normal and 5 abnormal groups. The synthetic ECG signal has a waveform pattern similar to the original ECG signal, the average RMSE between the two signals is 0.042 µV, and the average cosine similarity is 0.993. In addition, five classification models were developed to verify the effect of the synthetic dataset and showed performance similar to that of the model made with the actual dataset. In particular, even when the real dataset was applied as a test set to the classification model trained with the synthetic dataset, the classification performance of all models showed high accuracy (average accuracy 93.41%).

**Conclusion:**

The synthetic 12-lead ECG dataset was confirmed to perform similarly to the real-world 12-lead ECG in the classification model. This implies that a synthetic dataset can perform similarly to a real dataset in clinical research using AI. The synthetic dataset generation process in this study provides a way to overcome the medical data disclosure challenges constrained by privacy rights, a way to encourage open data policies, and contribute significantly to promoting cardiovascular disease research.

## Introduction

Recent advances in data generation and loading efficiencies and the development of computational science have encouraged researchers who have provided insightful studies in various academic fields [1, 2]. In the healthcare field, data that has been loaded for a long time worldwide is being constructed into a systematic database, like a common data model. This is influencing the rapid development of medical service technology using medical data along with the increase in demand for quality-of-life improvement [3]. Numerous large medical databases on diverse diseases, which are loaded over a long period of time, can be utilized for complex disease epidemiologic research and precise medical technology development according to the disease [4, 5].

Medical data are obtained in the form of electronic health records, medical images, biosignals, and comprehensive analyses of such data allow the diagnoses of diseases [6, 7]. Electrocardiogram (ECG) signals are representative medical data for the diagnosis of chronic cardiovascular diseases [8]. As an important risk indicator for cardiovascular diseases, ECG abnormalities are most commonly used in health informatics applications, such as disease prediction, classification, and telemedicine [9–11]. The 12-lead ECG is a gold standard in ECG testing that analyzes the heart’s activity states with 12 waveforms [12]. Cardiovascular disease is a high-risk disease that not only impairs a patient’s quality of life but also causes strokes or death, so continuous monitoring is required [13]. In addition, as a major symptom of COVID-19, the need for efficient preventive approaches to deal with cardiovascular disease by using the 12-lead ECG continues to increase [8, 14, 15]. For this reason, the demand for developing telemedicine services using 12-lead ECG and artificial intelligence (AI) continues to increase.

In 12-lead ECG studies, AI technology that can support the diagnosis of a clinician, such as classification and prediction of diseases, and regenerating missing ECG signals is being suggested [9, 16–19]. To overcome the limitations of AI technology applications for complex cardiovascular diseases that are difficult to develop through independent, small, or relatively homogeneous datasets, Alday et al. [9] held a cardiology challenge based on large 12-lead ECG databases published in PhysioNet. The characteristics of large publicly available 12-lead ECG databases were systematically analyzed to improve the utility of the database, and it contributed greatly to the study of cardiovascular disease classification by enabling more than development of 1000 AI algorithms. Attia et al. [16] developed an atrial fibrillation (AF) prediction model based on a large set of 12-lead ECG signals to prevent encephalopathy, heart failure, and death due to AF. For selecting training data in the AF prediction model, data recorded prior to the onset of atrial fibrillation (AF) was used. More than 1240,000 case records in clinical institutions from 1993 to 2017 were used. Their study is very meaningful in that it opened a new research direction for AF that could not be done without data accumulated for a long time. A number of studies have been conducted to regenerate the gold-standard 12-lead ECG using a reduced number of ECG leads for applications in remote patient management [17–19]. A large amount of public 12-lead databases was inevitably required to develop these AI models. However, there are many limitations in the development of artificial intelligence technology using 12-lead ECG data because an accurate database suitable for the conditions such as age, sex, and diagnosis required for the desired technology cannot exist.

Compared to other fields, big data in the medical field is difficult to access because parameters are more sensitive, and there is a limited number of open data sources that can be used for research [20, 21]. The same problem occurs with ECG signal data, and to overcome this problem, public ECG databases for research promotion are continuously released through collaborative work between general hospitals and universities [9, 22–25].

ECG data for public use is continuously released worldwide, there is a risk of data misuse and problems, such as a lack of incentives for data sharing [1, 26]. In addition, medical data must be reviewed by the institution’s complex deliberation procedures until disclosure, and there are countries where disclosure is restricted. As an approach to overcome this problem, a synthetic data generation method has been proposed that has characteristics of a real signal but does not need to be acquired in a clinical setting [27]. Synthetic data offers cost-effective alternatives to acquiring real-world data and circumvents legal, personal information, and policy restrictions, enabling the acquisition of large datasets for technology development [28, 29]. Previous studies have suggested extremely sensitive deep learning-based approaches for regeneration of ECG signal data. Golany et al. enhanced the accuracy of repetitive synthetic ECG signal data using an existing mathematical model-based ECG signal simulator and a Generative Adversarial Network (GAN) with Euler loss function [30]. Delaney et al. reconstructed ECG signals using a GAN model that utilizes Long Short-Term Memory (LSTM) [31]. However, existing studies using GAN have encountered significant issues in that the accuracy of the regenerated signal decreases with the quality of the training input, limiting its ability to accurately reflect the periodic cycle of 12-lead signals. In contrast, previous research that utilized peripheral signals to regenerate 12-lead ECG signals used leads 1, 2, and V2, which are most highly associated with the heart’s electrical activity, to recreate 12-lead ECG signals [17, 32]. However, each signal in the 12-lead ECG has unique combinations, correlated separately, and there is an accuracy deviation when regenerating signals using this fixed three-signal method [33].

The purpose of this study is to propose a novel approach to generating synthetic ECG signal datasets that can be used as public data. The dataset is constructed from a large-scale 12-lead electrocardiogram database in general hospitals and introduces a robust model for multiple diseases. In addition, a synthetic ECG signal is generating using the surrounding ECG signals most related to the electrical activity of a chosen ECG signal to construct a dataset. The clinical usefulness of the synthesized ECG dataset was evaluated by comparing the performance of five classification models trained with both synthetic and real datasets.

## Methods

### Design of the process for synthetic dataset generation

**Fig. 1:**
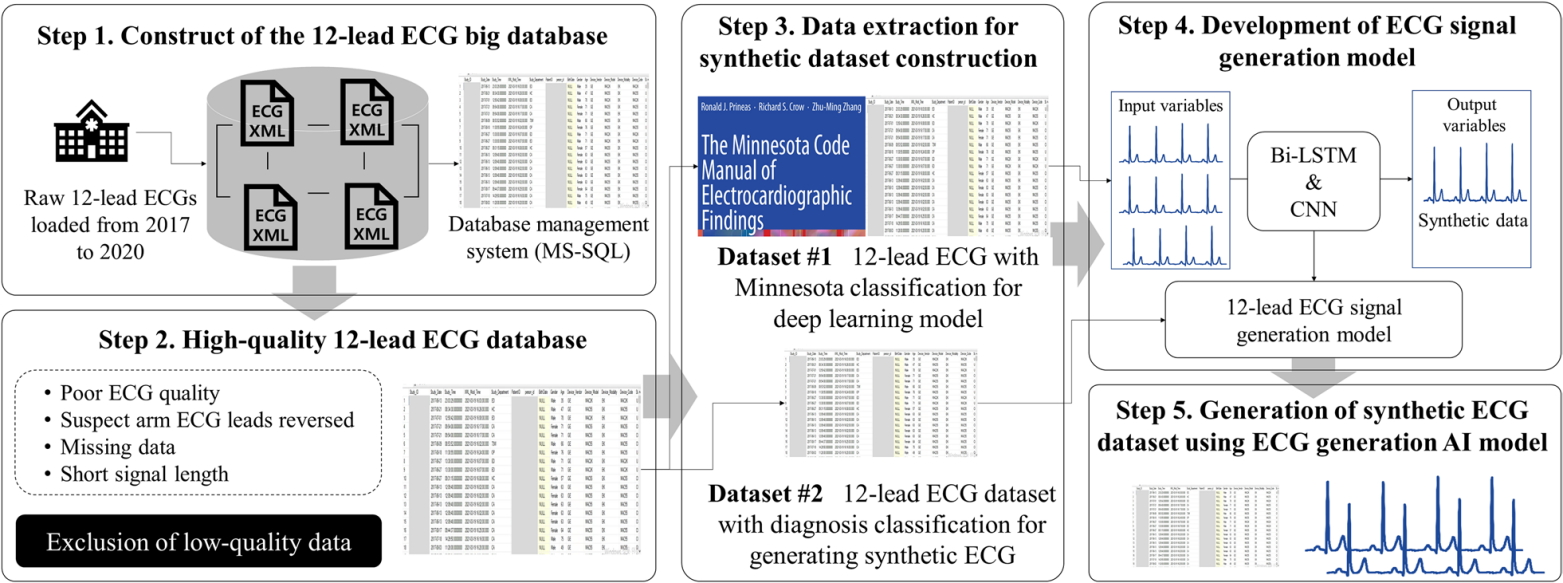
Proceeding steps for generating a synthetic 12-lead ECG signal dataset

The synthetic 12-lead ECG dataset construction process consists of a total of 5 steps, as shown in Fig. 1. In Step 1, raw 12-lead ECG data is collected from patients and loaded into the large database of a medical institution over a long period of time. In Step 2, a low-quality data removal step is performed to extract only high-quality data from the large 12-lead ECG database. In Step 3, using the filtered high-quality data, a training dataset for the ECG generation model and a dataset for generating a virtual dataset are built. In Step 4, a synthetic 12-lead ECG signal generation model is developed based on the training dataset and the AI model. Lastly, in Step 5, a synthetic ECG dataset is constructed by utilizing the developed ECG signal generation model from Step 4. Through the above process, based on the large, high-quality database owned by a medical institution, an open dataset that can be published in a large set without personal identification problems was built.

### Construction of a large 12-lead ECG database in a general hospital

The 12-lead ECG test was conducted for patients who visited the participating hospital, and the clinical expert directly input basic information into the hospital information system for analysis using General Electric (GE) and Philips equipment in the hospital settings. All 12-lead ECG were stored in a clinical information system (CIS, INFINITT Healthcare Co., Seoul, Korea) in eXtensible Markup Language (XML) file format. The standard 12-lead ECG data used in this study were obtained from the Clinical Information System (CIS) of Korea University Anam Hospital and included data from January 1, 2017, to December 31, 2020. The study protocol was approved by the institutional review board of the Korea University Anam Hospital (IRB NO. 2021AN0261). Written informed consent was waived because of the retrospective study design with minimal risk to participants. The study complied with the principles of the Declaration of Helsinki.

The ECG data is stored in XML format in the CIS server and includes the metadata of the patient’s basic personal information. The XML file contains basic examination information, technical data, eight ECG parameters, diagnosis statements, and waveform data. Basic examination information includes the patient registration number, examination date and time, and examination equipment, as well as technical data, including information such as the sampling rate, amplitude, and filtering frequency. The Python standard module (ElementTree XML API) was used to parse the data in the XML file of each ECG dataset, and all associated programming source code was written in Python 3.8.0. The data extracted from the XML file were loaded into a database management system (DBMS, Microsoft SQL server 2019 developer edition) that works with Python for effective data management. Data transfer to the DBMS is easily searchable complex condition data using SQL queries, and it can be combined with clinical chart data and managed conveniently. For the DBMS, MS-SQL was used, and the column type of the DBMS was set based on the data characteristic set. Through this process, the 12-lead ECG database loaded at the Korea University Anam Hospital was constructed.

### Datasets for developing a signal generation model and generating a synthetic dataset

In this study, two types of 12-lead ECG datasets were constructed. The first is a dataset for training an AI model for generating a synthetic signal, and the second is a dataset for generating a synthetic 12-lead ECG by applying it to the AI model. The ECG dataset was extracted from a 12-lead ECG database constructed with the DBMS. As a prior work to build two datasets, data of conditions that could include low-quality data were excluded. In order to use only high-quality data, the missing data in which the signal was measured as 0 µV by measuring with no electrode attached were excluded. In addition, ECG signals with insufficient frames caused by patient movement or electrode instability during the examination were excluded. Also, among the statements provided by the ECG machine, data including poor ECG quality and suspect arm ECG leads reversed, which can be classified as low quality, were excluded. In addition, only the data from the first visit was used to prevent data duplication caused by patients visiting regularly. Table 1 shows a list of statements of ECG machines that were used to exclude low-quality data.

**Table 1:** Exclusion criteria for the extraction of a high-quality dataset

Exclusion criteria	Vendor	Statement
Low-quality ECG	GE	Poor data quality, interpretation may be adversely affected
		Acquisition hardware fault prevents reliable analysis, carefully check ECG record before interpreting
		Baseline wander
		Current undetermined rhythm precludes rhythm comparison, needs review
		Electrode noise
		Muscle tremor
		Poor data quality
		Poor data quality in current ECG precludes serial comparison
	Phillips	All 12 leads are missing
		Artifact in lead(s)
		Artifact in lead(s) and baseline wander in lead(s)
		Baseline wander in lead(s)
		Incomplete analysis due to missing data in precordial lead(s)
		Missing lead(s)
		Missing lead(s) and partial lead(s)
		Poor-quality data—please repeat ECG!
Suspect arm ECG leads reversed	GE	Suspect arm lead reversal, interpretation assumes no reversal
		Arm lead reversal
	Phillips	Left arm and left leg electrode reversal
		Probable extremity electrode reversal
		Right and left arm electrode reversal
		Right arm and left leg electrode reversal

Two datasets were constructed to generate a synthetic 12-lead ECG dataset. The characteristic of the training dataset1 for developing the signal generation model is to extract it to include all ECG data for multiple diseases to generate both normal and patient ECGs The dataset classification criteria were based on the Minnesota classification system, and data were obtained from 2000 patients for each Minnesota classification [34]. The ECG machine provides around 147 diagnoses. Matching this to 10 Minnesota classifications was performed based on the clinical knowledge of a cardiologist. Table 2 shows three representative diagnoses for each Minnesota classification.

**Table 2:** Representative diagnosis according to Minnesota classification category

Minnesota classification category	Representative diagnosis
Unclassified	Sinus rhythm, Sinus arrhythmia, Prolonged QT interval
QRS axis deviation	Left axis deviation, Right axis deviation, Indeterminate axis
High amplitude R wave	LVH, RVH, Ventricular hypertrophy
Arrhythmia	Sinus rhythm (bradycardia), Atrial fibrillation, Sinus rhythm (tachycardia)
AV conduction defect	AV block, AV block (1st degree), PR interval (short)
Ventricular conduction defect	RBBB, RBBB (incomplete), rSr pattern in V1 and V2
Q and QS pattern	Myocardial infarction (inferior), Myocardial infarction (septal), Myocardial infarction (anterior)
ST junction and segment depression	Myocardial ischemia (lateral), ST-T abnormality (non-specific), Myocardial ischemia (anterior)
T wave items	T wave (abnormal), T wave (inverted), T wave (flattened)
Miscellaneous	ST segment elevation, P wave (abnormal), Voltage (decreased)

Minnesota classification was used, abbreviated, and slightly modified to reduce space

*AV* atrioventricular, *LAFB* left anterior fascicular block, *LVH* left ventricular hypertrophy, *RBBB* right bundle branch block, *RVH* right ventricular hypertrophy, *STEMI* ST segment elevation myocardial infarction

The dataset2 was constructed so that 6 diagnoses were extracted evenly. One normal diagnosis and 5 abnormal diagnoses were included, and the 5 abnormal diagnoses were selected to have different waveform patterns. 12-lead ECGs of 1000 patients were extracted for each diagnosis under the condition that does not overlap with dataset1, and a total of 6000 sets of data were obtained.

### Preprocessing of ECG signal

In general, a signal containing unnecessary noise is acquired due to various factors during ECG examination. To obtain diagnostic accuracy through monitoring, it is essential to remove the noise generated in the ECG signal. Butterworth filters are among the most commonly used signal-processing methods in the field of biomedical engineering [18, 35]. They provide an effective method to remove low-band signals generated from large movements and high-band signals caused by micro-electrode activation, such as muscle activity. In this study, the cut-off frequencies were set to obtain a range from 0.05 to 150 Hz to minimize the distortion of the ST-segment and to maintain the post-potential information of the QRS wave [18]. In addition, all 12-lead ECG data were converted between 0,1 by applying min–max normalization to utilize it as input data for AI models.

### Generating the synthetic ECG signals using the ECG signal generation model

**Fig. 2:**
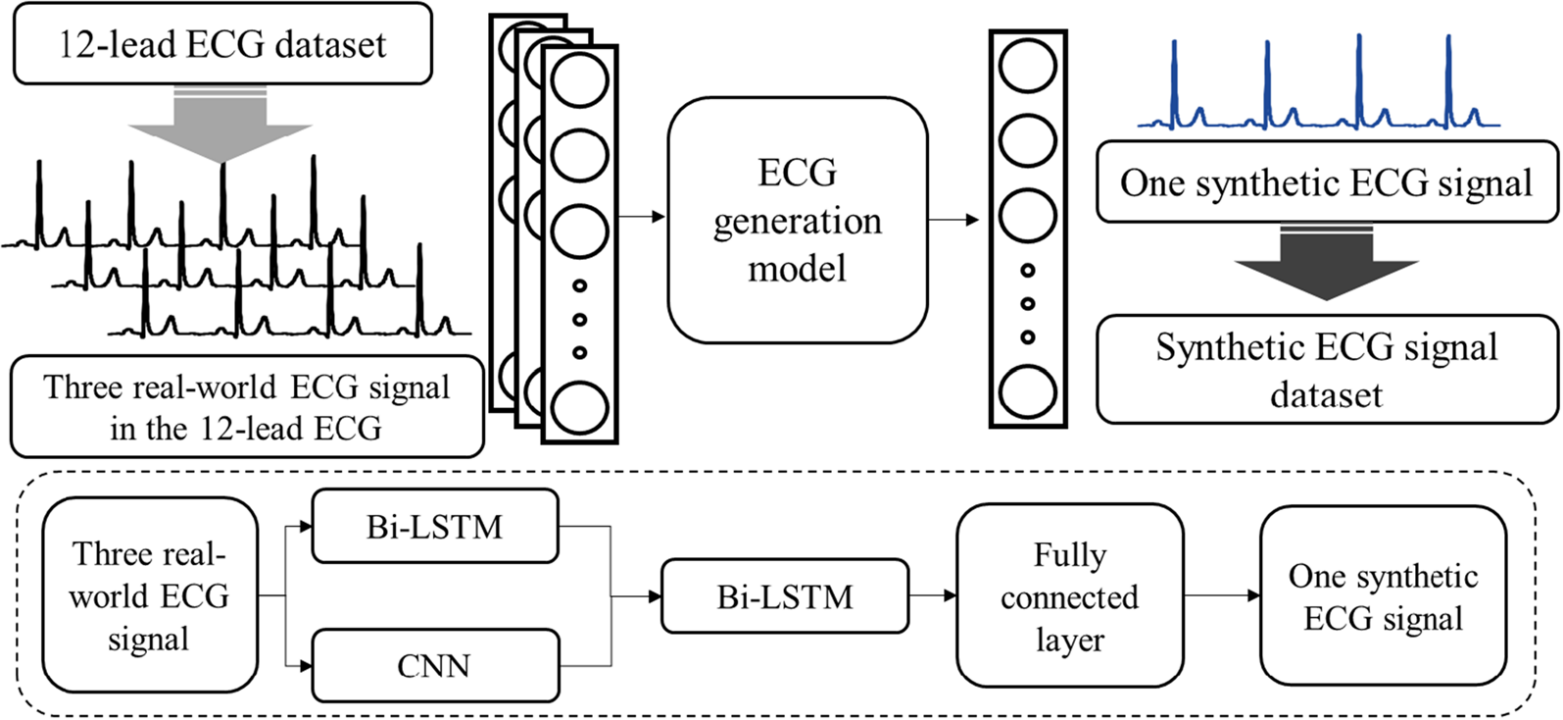
Overall process and AI model architecture for ECG generation

To generate a synthetic 12-lead ECG dataset, a deep learning model that can generate one synthetic ECG signal using three real-world ECG signals as input data was used. The architecture of the deep learning model for generating a synthetic ECG is shown in Fig. 2. The structure of the model for signal generation consists of two steps. First, we extract features from three signal data through bi-LSTM and CNN and combine them into one. And, in the second step, a signal is generated by using the previously combined features as input data to the bi-LSTM. In this study, models and results of studies for learning long frame data were cited [36–38]. According to previous studies, it has been reported that a model combining the LSTM and CNN models is better than the learning result using an LSTM and CNN alone, and it was reported that the bi-direction among the hyperparameters of the LSTM can give the best performance [38]. For this reason, a synthetic ECG dataset was generated using a model combined with the bi-LSTM and CNN models. The bi-LSTM structure for extracting the temporal features of the three signals was composed of four layers, and the number of nodes was set to 256, 128, 64, and 32. The CNN structure for extracting spatial features was composed of four 1-dimensional CNN layers, and the channels of each layer were set to 256, 128, 64, and 32. In addition, the bi-LSTM structure that generates one signal by combining two types of features is composed of two bi-LSTM layers and one fully connected layer. The activation function used was ReLU, and the learning rate was set to 0.0001. The input data was used in the form of a matrix of 5000 elements by 3 by combining signals composed of 5000 frames into one. Also, the output data was set in the form of 5000 by 1 to acquire one signal composed of 5000 frames.

A combination of three signals to generate one signal was set based on the results of a previous study. In the previous study, to obtain an optimal signal combination, 11 signals, except for the signal to be regenerated, were combined 3 each, and the combination showing the best performance was selected as input data by learning them through linear regression [33]. The optimal combination selected for each signal is shown in Table 3. The combination was used as input data for the synthetic dataset generation model. The accuracy of the generated synthetic ECG signal was analyzed by calculating the root mean square error (RMSE) and cosine similarity of the real-world ECG signal. The RMSE and cosine similarity analysis between the synthetic ECG signal and the real-world ECG signal were calculated according to the lead and diagnosis classification. Cosine similarity is a measure used to quantify the similarity between two vectors by calculating the cosine. A cosine similarity value closer to 1 indicates a higher similarity, indicating that the two vectors have a similar direction.

**Table 3:** Combination of input signals for training the ECG generation model

Combination of input signal					
Standard leads			Extremity leads		
Lead1	Lead2	Lead3	aVR	aVL	aVF
aVR, aVL, V6	aVR, aVF, V6	Lead2, aVL, aVF	Lead1, Lead2, V6	Lead1, Lead3, aVR	Lead2, Lead3, aVR

Precardiac leads					
V1	V2	V3	V4	V5	V6
Lead1, aVR, V2	V1, V3, V4	V2, V4, V5	V2, V3, V5	V3, V4, V6	aVR, V4, V5

### Technical validation of synthetic ECG dataset using classification model

**Fig. 3:**
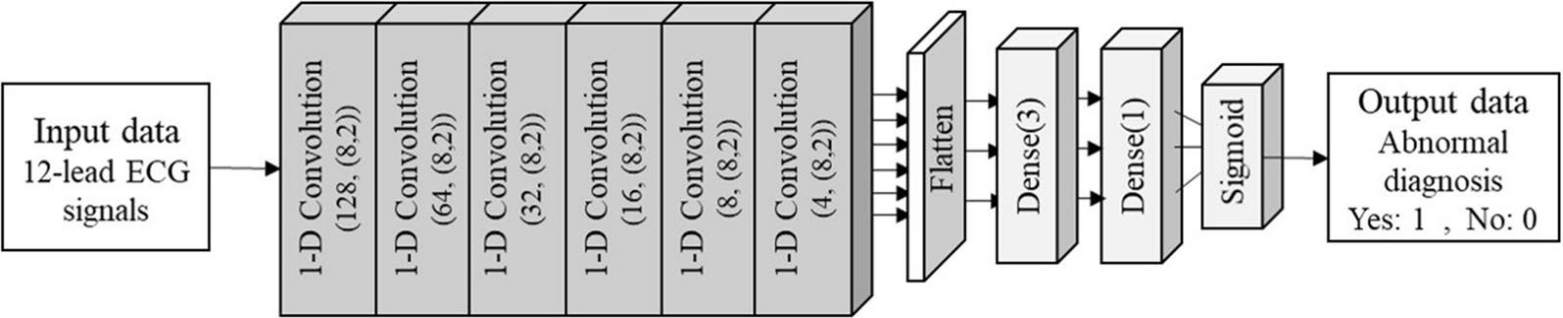
Architecture of the abnormal ECG signal classification model

In order to verify whether the synthetic 12-lead ECG dataset has clinical usefulness as a dataset for facilitating AI research, an abnormality classification model of the real dataset and the classification model of the synthetic dataset were developed and analyzed. For the abnormality classification model, a total of 10 models were developed: 5 models trained from the real dataset obtained for the purpose of generating a synthetic dataset, and 5 models trained from the dataset generated through the signal generation model. The dataset used in each model consists of 1000 normal datasets and 1000 abnormal datasets. The input data of all classification models used all 12-lead ECG signals. As shown in Fig. 3, the model architecture consists of 6 1-D CNN layers and 2 dense layers. The training, validation, and test data sizes were applied with the respective ratios of 6:2:2, and the accuracy of the model was evaluated through fivefold cross-validation (CV). The learning rate of the model was established as 0.001. The accuracy, F1-score, recall, and precision of the two models trained with the real-world dataset and the synthetic dataset were analyzed through the independent t-test. The statistical significance level was set at *p* < 0.01. All statistical analyses were performed using SPSS 15.0 software (SPSS Inc., Chicago, IL, USA). In addition, the model trained with the synthetic dataset was verified by using twenty percent of the real-world dataset as a test set for the performance of the model. The performances of the models were quantitatively confirmed through the accuracy, F1-score, recall, and precision.

## Results

### Data selection and preprocessing of signal

The results of the three steps to exclude low-quality datasets before obtaining two high-quality datasets are as follows. In the first step, ECG cases of the ECG statements that the data has poor ECG quality and a suspicious arm ECG lead were removed from the source data (n = 286,542). Second, ECG cases containing missing data in any ECG leads and a frame count of less than 5000 were also excluded (n = 7644). In the third step, ECG data from the first visit of a patient to a hospital were selected from multiple ECG cases of the same patient (n = 157,594).

The details of the two datasets obtained using a high-quality database are shown in Table 4. To develop the signal generation model, 2000 ECG datasets (one ECG per subject) were selected from each of the 10 Minnesota categories out of 157,594 ECG cases. For the synthetic dataset, 1000 ECG datasets (one ECG per subject) were selected from each of the 6 diagnosis categories among the ECG cases, excluding the data from dataset1 in the high-quality 12-lead ECG database.

**Table 4:** Characteristics of datasets

	Dataset1	Dataset2
Signals (n)	22,000	6000
Original frequency (Hz)	500	
Recording time (s)	10	
Leads (n)	12	
classification criteria	Minnesota	Diagnosis
Categories (n)	10	6
Detailed category	Unclassified	Normal sinus rhythm
	QRS axis deviation	Sinus bradycardia
	High amplitude R wave	Left axis deviation
	Arrhythmia	Atrial fibrillation
	AV conduction defect	First-degree atrioventricular block
	Ventricular conduction defect	
	Q and QS pattern	Prolonged QT interval
	ST junction and segment depression	
	T wave items	
	Miscellaneous	

### Results of the generation a synthetic 12-lead ECG dataset

**Fig. 4:**
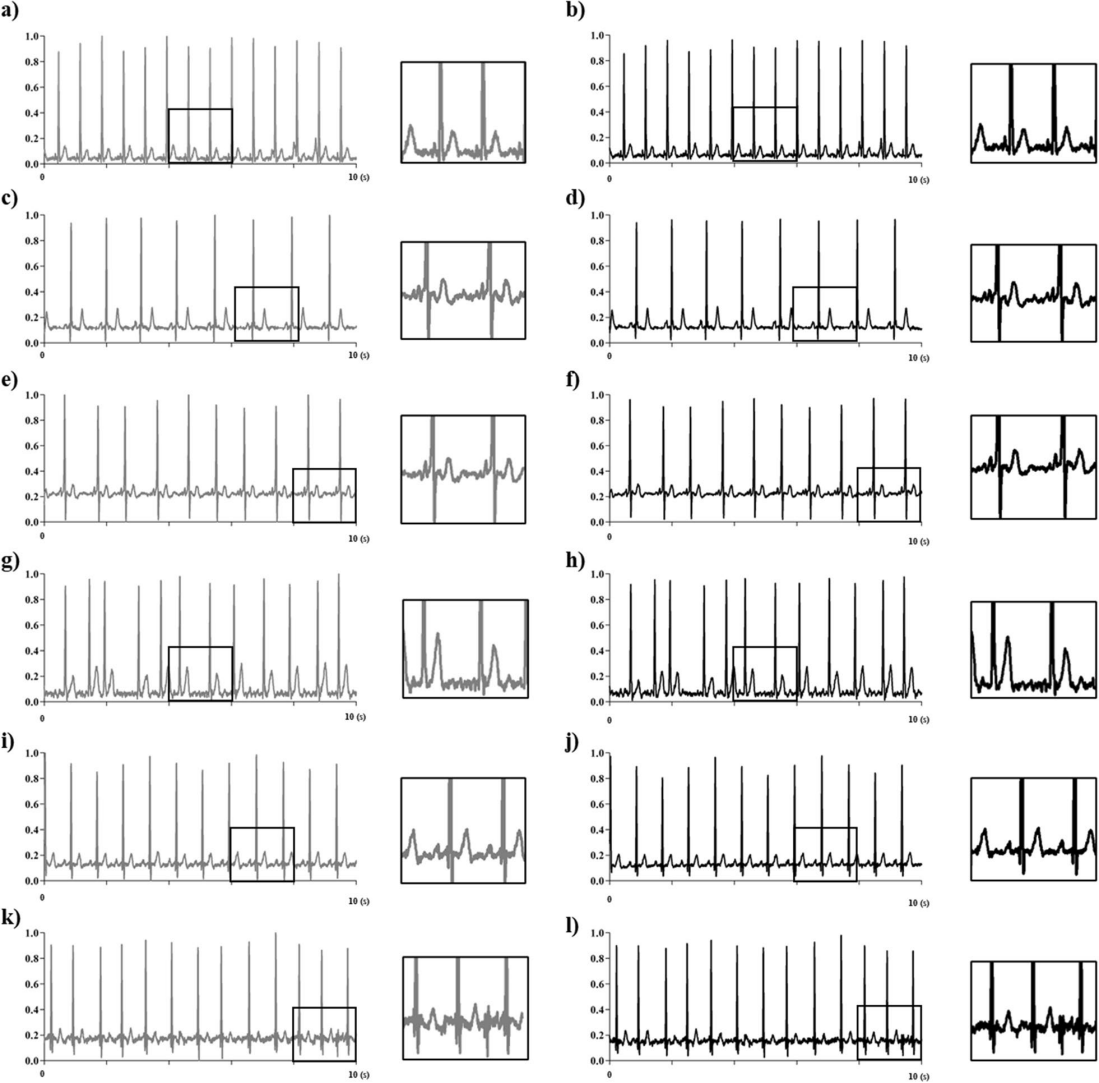
Real-world ECG signal and synthetic ECG signal on lead 2 (the gray line represents real-world ECG data and the black line is the mean synthetic ECG; **a**, **b** Normal sinus rhythm; **c**, **d** Sinus bradycardia; **e**, **f** Left axis deviation; **g**, **h** Atrial fibrillation; **i**, **j** First degree atrioventricular; **k**, **l** Prolonged QT interval)

A synthetic 12-lead ECG dataset was generated with a total of 12 AI models. As a result of model validation using the test set, the average root mean square error (RMSE) of all models was 0.038 µV, indicating high generation accuracy. The synthetic dataset generated in this study consisted of thousands of 12-lead ECG for a total of six categories of statements. Figure 4 shows the real-world ECG data and the synthetic ECG data generated by the model. The gray line and black line mean real-world ECG signal and synthetic ECG signal, respectively. The synthetic signal showed an overall similar morphology, characteristic peaks, and temporal intervals as the real signal. The synthetic ECG dataset consists of a total of 6000 by generating 1000 for each diagnosis category, and all signals are composed of 5000 frames, like the real signal for 10 s at 500 Hz.

Table 5 is the result of the RMSE of the synthetic dataset compared with the real-world dataset. As a result of calculated the RMSE to verify the quality of the synthetic ECG dataset, the average RMSE of all ECG signals was 0.043 µV. The RMSE according to diagnosis showed the lowest error with an average of 0.034 µV for the normal sinus rhythm, and the highest error with an average of 0.052 µV in a first-degree atrioventricular block. As a result of analysis by lead, lead 3 showed the lowest RMSE and v1 showed the highest RMSE. In particular, compared to other leads, Lead 1, Lead 2, and Lead 3, belonging to the standard lead, showed lower RMSEs. The average RMSEs for standard leads, extremity leads, and precardiac leads were 0.0297, 0.0341, and 0.0532 µV, respectively.

**Table 5:** RMSEs of synthetic dataset compared with the real-world dataset for each ECG signals

	Normal sinus rhythm	Sinus bradycardia	Left axis deviation	Atrial fibrillation	First-degree atrioventricular block	Prolonged QT interval	Mean ± SD (µV)
Lead 1	0.026	0.028	0.039	0.041	0.028	0.049	0.035 ± 0.009
Lead 2	0.016	0.017	0.028	0.024	0.020	0.028	0.022 ± 0.005
Lead 3	0.026	0.026	0.031	0.035	0.034	0.038	0.032 ± 0.005
aVR	0.028	0.029	0.053	0.035	0.032	0.036	0.036 ± 0.009
aVL	0.031	0.036	0.043	0.047	0.043	0.065	0.044 ± 0.012
aVF	0.014	0.017	0.022	0.023	0.023	0.036	0.023 ± 0.008
V1	0.062	0.063	0.083	0.079	0.071	0.088	0.076 ± 0.009
V2	0.041	0.042	0.048	0.052	0.043	0.053	0.047 ± 0.005
V3	0.049	0.051	0.047	0.054	0.051	0.063	0.052 ± 0.006
V4	0.040	0.042	0.045	0.048	0.043	0.051	0.045 ± 0.004
V5	0.034	0.030	0.045	0.047	0.040	0.049	0.041 ± 0.008
V6	0.051	0.045	0.065	0.064	0.053	0.071	0.058 ± 0.010

*SD* standard deviation

### Construction of a large 12-lead ECG evaluation of synthetic dataset using the classification models

To verify the synthetic ECG dataset, a total of 10 models trained on the real-world dataset and the synthetic dataset were developed. Both types of models for sinus bradycardia, atrial fibrillation, and first-degree atrioventricular block showed more than 90% accuracy, and more than 80% accuracy for other diagnoses (Table 6). In particular, it was confirmed that high prediction accuracy was secured in the model for the diagnosis of sinus bradycardia and atrial fibrillation. The classification performance of the models using actual and synthetic data varied slightly depending on the diagnosis category. However, no significant difference was found in the statistical analysis between the two models using synthetic and real-world ECG datasets.

**Table 6:** Performance of classification models trained with a real-world dataset and synthetic dataset in accordance with diagnosis through fivefold cross-validation

Categories	Accuracy (%)		Precision		Recall		F1-score	
	Orig	Syn	Orig	Syn	Orig	Syn	Orig	Syn
Sinus bradycardia	97.70	97.02	0.98	0.95	0.97	0.99	0.98	0.97
*p* value	0.29		0.25		0.50		0.19	
Left axis deviation	85.00	84.45	0.87	0.85	0.78	0.82	0.84	0.84
*p* value	0.39		0.07		0.22		0.47	
Atrial fibrillation	97.50	96.34	0.94	0.96	0.99	0.97	0.97	0.97
*p* value	0.13		0.15		0.12		0.28	
First-degree atrioventricular block	90.74	91.01	0.93	0.91	0.87	0.91	0.91	0.91
*p* value	0.43		0.09		0.14		0.41	
Prolonged QT interval	81.60	82.16	0.90	0.89	0.71	0.71	0.79	0.79
*p* value	0.34		0.20		0.48		0.41	

Additionally, the clinical usefulness was verified by applying the real-world dataset as a test set to the model trained on the synthetic dataset. As a result of using the real dataset as the test set for all models trained with synthetic data, similar performance was shown compared to the model trained with the real dataset, as shown in Table 7. This an accuracy of over 90% in sinus bradycardia, atrial fibrillation, and first-degree atrioventricular block, and over 88% for the remaining two diagnoses. In addition, as a result of evaluating the area under the curve (AUC) of the model trained with a synthetic dataset derived from a real-world dataset, the average of the five models was 0.97, indicating high classification performance.

**Table 7:** Performance of classification models by applying the real-world dataset as a test set to the model trained with the synthetic dataset

Categories	Test set	Accuracy (%)	Precision	Recall	F1-score
Sinus bradycardia	Real-world	98.13	0.98	0.99	0.99
Left axis deviation	Real-world	89.23	0.80	0.96	0.87
Atrial fibrillation	Real-world	98.63	0.99	0.99	0.99
First degree atrioventricular block	Real-world	92.96	0.89	0.97	0.93
Prolonged QT interval	Real-world	88.09	0.88	0.80	0.84

## Discussion

A synthetic 12-lead ECG dataset was created to build an open database that is not restricted by personal information and public data policies. The synthetic ECG signal was built using data obtained with the 12-lead ECG machines of GE and Philips, which were used in a general hospital, to create clinically useful data. The generated 12-lead ECG dataset is divided into six categories, including normal and abnormal diagnosis categories a total of 6000 12-lead ECG signals, each composed of 5000 frames, are included. The advantage of the synthetic dataset produced by this study is that it generates a data pattern almost similar to real-world signals by using a high-quality generation model based on a large 12-lead ECG database acquired for 10 s at 500 Hz in a general hospital. The synthetic ECGs generated in this study showed high accuracy in most of all signals of a 12-lead ECG. However, compared to other signals, V1 showed a relatively high, though still modest, RMSE. It is considered that the reason for this is that the optimal combination of the input data of the model that generates V1 is extracted one by one from the three lead systems. In the original dataset used in this study, it is difficult to analyze the relationship between signals due to various disease groups. The optimal combination of V1 was only V2 from the same lead system and the rest were obtained from other lead systems. Although it was an optimal combination selected by analyzing all combinations, it is judged that the feature parameters for generating the synthetic V1 signal were slightly insufficient. However, the synthetic dataset showed an average RMSE of 0.043 ± 0.02 µV compared with the real-world dataset, and the accuracy of the two models developed through the real and synthetic datasets was not statistically different. This result implies that the signal characteristics of the diagnosis can be sufficiently reflected by maintaining the overall pattern of the signal even though there was a slight mismatch in amplitude. In addition, the results of validating the model trained with the synthetic dataset with the original dataset showed high accuracy in all diagnostic classification models. To sum up the results, the proposed synthetic dataset can be used as open data in the field of AI research instead of an original dataset comprised of protected personal data.

The synthetic dataset has a low possibility of identifying patient information and has the advantage that it can be used as a method to construct a database suitable for a specific research purpose. The reason that it is difficult to disclose medical data is that it contains personal information such as the date of birth, name, and address, and the patient can be identified through clinical data [4]. However, the generated synthetic 12-lead ECG dataset provides only signal data and patient data is not identifiable because the signals are normalized to the maximum and minimum values to facilitate AI training. Since the min–max values of the original signal are unknown to anyone other than the person in charge of generating the synthetic dataset, the real signal cannot be traced unless the exact min and max values are known. Even if pattern analysis is performed through the correlation coefficient, the identification probability is low because a high correlation coefficient is obtained in the same disease group. Furthermore, dataset construction through synthetic signals has the advantage that one is able to construct a large and open database that suits the research purposes because it can be built based on a real-world database where the patterns of all the diseases exist. A practical limitation of the existing publicly available ECG databases is that the data characteristics are different because data were obtained from various commercial machines. This means that it is difficult to combine databases and apply to big data and AI research. Also, public databases consist of as few as one to as many as twenty or more subclasses may cause a data biasing problem, and the bias will reduce the accuracy of the AI model [1, 39].

It is difficult to disclose high-quality real-world ECG databases due to de-identification and data policy requirements. Research using medical data can be conducted by performing IRB procedures in accordance with the policies of medical institutions, but there are many administrative difficulties in converting to public data and disclosing it for the purposes of promoting domestic and foreign research [40]. Previous studies have been trying to provide usable public open data on various diseases using open platforms. The authors also tried to convert the synthetic dataset to open data by applying a high-quality 12-lead ECG database extraction method from a large database of a general hospital to promote further AI and machine-learning research. However, in a previous study, the constructed dataset could not be disclosed due to various policy problems and only the database construction process was provided [24]. Since the method of this study reflects only the clinical ECG signal pattern of the disease and does not provide real-world data values, it will be able to overcome the problem of converting private patient data to public synthetic open data.

It has been confirmed that the synthetic dataset generated exhibits similar performance to the real-world dataset through the development of diagnosis classification models. However, synthetic datasets have some limitations. First, in this study, based on the international standard OMOP-CDM, a cardiologist standardized diagnoses that have multiple names, and a synthetic ECG signal was constructed using this standardized ECG dataset. Although the overall accuracy of computerized ECG interpretation has been reported to be 88%, further validation by a cardiologist is needed with the synthetic 12-lead ECG to minimize the potential for diagnostic errors in clinical applications. Second, it is difficult to apply the synthetic dataset to clinical research using ECG peaks because the amplitude of the synthesized ECG signal is different from that of the actual ECG signal. Third, the scope of the study was limited because there was no electronic health record information available corresponding to the synthetic 12-lead ECG. For this reason, additional complex studies, especially those aiming to predict clinical outcomes or make more nuanced clinical decisions, that consider patient characteristics are necessary. This could include demographic information (like age or gender), clinical indicators (like heart rate, blood pressure, cholesterol levels), medical history, medication use, and lifestyle factors (like smoking and alcohol consumption). Future studies securing and linking such additional clinical data, should consider (1) a method of combining ECG data and clinical data as synthetic 12-lead ECG data. In addition, (2) a method of anonymizing clinical data and linking it with synthetic 12-lead ECG data should be considered. Although the scope of the research was restricted due to the limitations, a synthetic ECG dataset could be generated based on a large 12-lead ECGs by setting conditions such as diagnosis, age, gender, and duration according to the purpose of the research. It is expected that our method that generates a large synthetic ECG dataset can be effectively used for cardiovascular disease classification and AI prediction and classification research.

## Conclusion

In this study, for the purpose of facilitating AI research on cardiovascular diseases, a synthetic ECG dataset construction process was established using a large set of 12-lead ECGs from a general hospital. The synthetic 12-lead ECG dataset consists of normal ECGs and five types of abnormal ECG signals, with a total of 6000 synthetic ECGs. The ECG signal generated by the model showed a waveform pattern similar to that of a real-world ECG. Furthermore, in the five classification models using synthetic and real datasets, the two models showed high classification accuracy and no statistical difference. This means that the synthetic ECG dataset can be used for AI research in cardiovascular disease without risking patient privacy.

Since the synthetic signal is generated based on the correlations between real signals, a small amplitude error occurs. Because it is synthetic data, it is not possible to perform extended research combined with other medical data. However, since the synthetic dataset was generated based on a real dataset, most of the waveform patterns of the disease are maintained. There is also the advantage of reducing the possibility of release of personally identifiable information in the process of generating synthetic data. Generating a synthetic 12-lead ECG dataset based on a large set of 12-lead ECGs is one way to allow efficient utilization of a high-quality dataset. This synthetic ECG dataset is expected to make a significant contribution to the work of many researchers interested in studying cardiovascular disease through AI.

## Data Availability

The datasets generated during and/or analyzed during the current study are available from the corresponding author on reasonable request.

## Abbreviations

ECG: Electrocardiogram
AI: Artificial intelligence
CNN: Convolution neural network
Bi-LSTM: Bi-directional long short-term memory
RMSE: Root mean square error
CIS: Clinical information system
XML: eXtensible Markup Language
DBMS: Database management system
CV: Cross-validation
IRB: Institutional Review Board
